# WMGHMDA: a novel weighted meta-graph-based model for predicting human microbe-disease association on heterogeneous information network

**DOI:** 10.1186/s12859-019-3066-0

**Published:** 2019-11-01

**Authors:** Yahui Long, Jiawei Luo

**Affiliations:** College of Computer Science and Electronic Engineering, Hunan University, Changsha, 410082 China

**Keywords:** Microbe, Disease, Association prediction, Weighted meta-graph, Heterogeneous information network

## Abstract

**Background:**

An increasing number of biological and clinical evidences have indicated that the microorganisms significantly get involved in the pathological mechanism of extensive varieties of complex human diseases. Inferring potential related microbes for diseases can not only promote disease prevention, diagnosis and treatment, but also provide valuable information for drug development. Considering that experimental methods are expensive and time-consuming, developing computational methods is an alternative choice. However, most of existing methods are biased towards well-characterized diseases and microbes. Furthermore, existing computational methods are limited in predicting potential microbes for new diseases.

**Results:**

Here, we developed a novel computational model to predict potential human microbe-disease associations (MDAs) based on Weighted Meta-Graph (WMGHMDA). We first constructed a heterogeneous information network (HIN) by combining the integrated microbe similarity network, the integrated disease similarity network and the known microbe-disease bipartite network. And then, we implemented iteratively pre-designed Weighted Meta-Graph search algorithm on the HIN to uncover possible microbe-disease pairs by cumulating the contribution values of weighted meta-graphs to the pairs as their probability scores. Depending on contribution potential, we described the contribution degree of different types of meta-graphs to a microbe-disease pair with bias rating. Meta-graph with higher bias rating will be assigned greater weight value when calculating probability scores.

**Conclusions:**

The experimental results showed that WMGHMDA outperformed some state-of-the-art methods with average AUCs of 0.9288, 0.9068 ±0.0031 in global leave-one-out cross validation (LOOCV) and 5-fold cross validation (5-fold CV), respectively. In the case studies, 9, 19, 37 and 10, 20, 45 out of top-10, 20, 50 candidate microbes were manually verified by previous reports for asthma and inflammatory bowel disease (IBD), respectively. Furthermore, three common human diseases (Crohn’s disease, Liver cirrhosis, Type 1 diabetes) were adopted to demonstrate that WMGHMDA could be efficiently applied to make predictions for new diseases. In summary, WMGHMDA has a high potential in predicting microbe-disease associations.

## Background

Accumulating clinic evidences have shown that the microbes residing in human hosts play a crucial role in the pathological mechanism of an extensive variety of human diseases. The microorganisms reside in and on human body with a wide range of organs like lung, skin, oral cavity and gut, most of which reside in gastrointestinal tract [1]. A plenty of experimental results have shown that most of commensal microbial communities benefit human health, and are even indispensable for human physiology because they could not only offer protection from pathogens and promote metablic capability, but also assist modulation of gastrointestinal development [2]. It is reported that there exist about 10^14^ microorganism cells inhabiting an adult intestine, which is approximately 10 times more than human cells [3]. These cells could produce a large amount of gene product which is essential for various metabolic and biochemical activities [4, 5]. Therefore, human microbes are also often treated as a forgotten organ of human due to its similar metabolic capacity to the liver [6]. Previous studies discovered that the microbial communities were significantly affected by the genetics [7–9] as well as the dynamic habitat environments, such as season [10], smoking [11], diets [12] and antibiotics [13]. The dynamic changes of these factors can lead to the imbalance of microbial communities and further affect the biological progress (i.e., metabolism, proteomic) of associated microbes, which possibly motivates a variety of important human diseases, such as asthma [14], diabetes [15], liver diseases [16], and even cancer [17].

Since the first microorganism which can cause human disease was found in the 1800s, an increasing number of microorganisms have been demonstrated to be the causation of different human diseases. For example, in order to determine the relationship between the clinical features of asthma and the composition of the airway bacterial microbiota, Huang et al. [18] utilized culture-independent tools to detect the relative abundance and presence of most known bacteria. As a result, they demonstrated that there existed closely relevant associations between the relative abundance of members of the *Comamonadaceae*, *Sphingomonadaceae*, *Oxalobacteraceae* and the degree of bronchial hyperresponsiveness. Larsen et al. [19] studied the differences between the composition of the intestinal microbiota in humans with type 2 diabetes and non-diabetic persons as control and found the compositional changes of intestinal microbiota like *Firmicutes*, *Lactobaillus*, *Bacteroidetes*, *Bacilli* and *Proteobacteria*. Moore et al. [20] analyzed the fecal floras from five diverse polyp patients including North American Causasians, Japanese-Hawaiians, rural native Africans and rural native Japanese. They eventually found the positive associations between increased risk of colon cancer and *Bacteroides* species and *Bifidobacterium* species while the closest associations of some *Lactobacillus* species and *Eubacterium* aerofacients with low risk of colon cancer. Identifying candidate microbes for diseases could not only offer insight into the pathological mechanism of human diseases, but also promote disease prevention, diagnosis, treatment and prognosis [21]. Even though the roles microbes play in the mechanism of human diseases have been increasingly revealed, a comprehensive understanding of microorganism remains largely challenge.

Considering that traditional experimental methods which researchers used to heavily depending on are time-consuming, expensive and laborious, researchers paid more attention to the development of computational methods for exploring microbe-disease associations (MDAs). A number of existing methods are implemented based on HIN consisting of multiple biological networks, such as KATAHMDA [22], RWRHMDA [23], NTSHMDA [24], PBHMDA [25]. For example, Chen et al. [22] proposed the first computational model of KATZHMDA to infer latent MDAs on HIN. In this model, all microbe-disease relationship pairs are prioritized according to their probability scores obtained by calculating the numbers of walks with different lengths between microbe nodes and disease nodes. However, it is possible for this model to cause bias towards well-investigated diseases and microbes. Shen et al.[23] implemented random walk with restart (RWR) on the HIN to prioritize candidate microbe for diseases. Unfortunately, since ignoring the bias rating of tendency to be associated with different neighbor microbe nodes for disease nodes, this method fails to achieve desired prediction performance. To overcome this challenge, following it, Luo et al.[24] proposed another model NTSHMDA, which utilizes extended RWR optimized by introducing network topological similarity to rank candidate microbes for diseases. Nevertheless, the aforementioned methods only consider Gaussian kernel interaction profile similarity to calculate similarity for both disease and microbe, yet ignore rich prior information on diseases and microbes. To take advantage of such information, researchers have recently paid more attention on prioritizing disease-associated microbes by incorporating prior biological knowledge [26, 27]. As an instance, Huang et al. [26] released a microbe prioritization method, which combines two single prediction models, namely neighbor-based collaborative filtering and graph-based scoring method. This method introduces symptom-based disease similarity to improve the completeness of disease similarity. Zhang et al. [27] presented a label propagation-based method of BDSILP for ranking candidate microbes for diseases, which incorporates multiple similarities for diseases and microbes, such as disease semantic similarity and microbe functional similarity. However, for the aforementioned methods, although integrating external biological information about diseases and microbes into prediction model, they still fail to make predictions for new disease without any known associations.

Recently, machine learning has been applied in the bioinformatics and computational biology, such as miRNA-target association prediction [28], lncRNA-disease association prediction [29, 30], drug combination prediction [31], miRNA-disease association prediction [32–34] and miRNA regulatory module identification [35]. A large number of machine learning-based algorithms have been also proposed for inferring MDAs [36–38]. For example, Wang et al. [36] developed a semi-supervised computational model of LRLSHMDA, which uses Laplacian regularized least squares classifier to prioritize disease-related microbes.

In this work, we proposed a novel computational model of WMGHMDA for inferring candidate microbes for diseases on HIN based on Weighted Meta-Graph. This model incorporates multiple sources of prior biological knowledge and could be applied to make predictions for new diseases without any known associations. Our approach involves three steps. First, a HIN is constructed by connecting the integrated microbe and disease similarity networks via observed microbe-disease bipartite network. Next, a pre-designed Weighted Meta-Graph search algorithm is iteratively implemented on the HIN to enumerate weighted meta-graphs related to each microbe-disease pair. Finally, we calculate the probability score for each microbe-disease pair by summing up the contribution values of relevant weighted meta-graphs and prioritize candidate microbes for diseases according to their probability scores. We carried out comprehensive experiments to evaluate the prediction performance of our method and demonstrated the improvement of prediction accuracy compared to state-of-the-art methods. In particular, WMGHMDA is capable of recovering average 75.4% of known true positive samples in the top-100 prediction for three complex human diseases.

Mainly, our contributions are as follows:

- We propose a novel computational model of WMGHMDA for predicting MDAs, which is the new application of meta-graph. To our knowledge, WMGHMDA is the first tool to use weighted meta-graph for microbe-disease association prediction.
- To improve the completeness of similarity, multiple prior biological knowledge is introduced in this paper, including disease semantic similarity and microbe functional similarity, which effectively boosts the improvement of prediction accuracy.
- In the network, subtle semantics between diseases and microbes are prolifically hidden. To capture this feature, we generalize common unweighted meta-graph to weighted meta-graph based on which we design a novel Weighted Meta-Graph search algorithm and leverage it to prioritize candidate microbes for diseases.
- Comparisons with state-of-the-art methods on HMDAD demonstrate the superiority of our approach. In addition, the approach is particular effective for a new disease with few known related microbes or without any known related microbes.

### Related work

Recently, a large number of tools have been developed for identifying MDAs. Most of existing methods are based on the assumption proposed by Ma et al. [21] that the functionally similar microbes tend to present interaction or non-interaction with phenotypically similar diseases and vice versa.

Predicting MDAs based on network analysis has become popular [22, 25, 27, 39, 40]. These methods attempt to infer the possibility of existing associations between diseases and microbes through HIN consisting of different biological networks. For example, Chen et al. [22] proposed a computational model of KATZHMDA based on HIN. This model infers potential association pairs using KATZ measurement on the network. Huang et al. [25] leveraged a special depth-first search framework on HIN for predicting candidate microbes for diseases. However, such methods calculate the similarities for both diseases and microbes strongly depending on Gaussian kernel similarity, which, as a result, tends to “recommend” well-studies microbes with more known associated diseases. In contrast, our proposed method combines multiple prior knowledge and alleviates this problem.

Random walk has been applied for prioritizing candidate microbes for diseases [23, 24, 41, 42]. Most of these methods are developed based on RWR, the variant of random walk. They make full use of the advantage of RWR in capturing local and global network topological characteristics. As an instance, Shen et al. [23] utilized extended random walk to uncover disease-related microbes but failed to consider the bias rating for different association pairs. To tackle such problem, Luo et al. [24] further improved this model by introducing network topological similarity. Unfortunately, these methods are limited in inferring possible microbes for diseases with few known associated microbes or without any known associated microbes. In this paper, our method is able to make predictions for new diseases by applying weighted meta-graph that could identify hidden subtle semantic relations between a disease and considered microbes only if the similarity feature can be obtained for the disease.

More and more attention has been recently paid to the application of machine learning in the prediction of MDAs [36–38]. Most of these methods achieves the prediction based on matrix factorization. For example, Shen et al. [38] introduced collaborative matrix factorization to update the correlation matrix of diseases and microbes for ranking candidate microbes for diseases. He et al. [37] released a novel computational methods of GRNMFHMDA based on graph regularized non-negative matrix factorization, but the selection of optimal parameters for this method remains a challenge. In addition, Wang et al. [36] proposed a semi-supervised computational model of LRLSHMDA, which uses Laplacian regularized least squares classifier to prioritize disease-related microbes.

## Results

### Performance evaluation

**Fig. 1:**
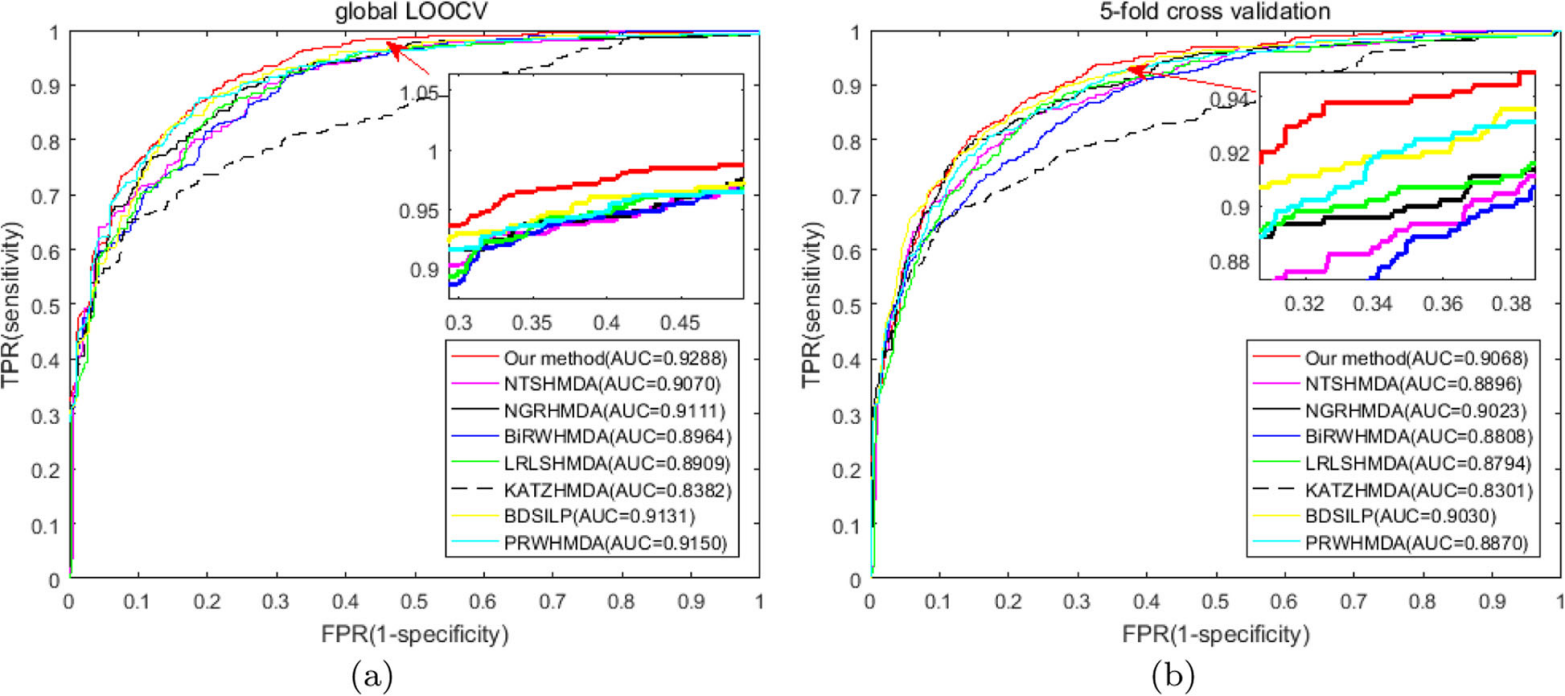
Comparisons of prediction performance between our method and five state-of-art prediction models (NTSHMDA, NGRHMDA, BiRWHMDA, LRLSHMDA, KATZHMDA, BDSILP and PRWHMDA) in the frameworks of global LOOCV and 5-fold CV, respectively. **a** The ROC curves and AUC values based on LOOCV, **b** The ROC curves and AUC values based on 5-fold CV

In order to measure the prediction accuracy of the proposed WMGHMDA model, we implement global LOOCV and 5-fold CV on HMDAD, respectively. In the framework of LOOCV, each observed microbe-disease pair is selected as test sample in turn while the rest observed microbe-disease association pairs are considered as training samples. In each round, the test sample is ranked according to its prediction score against all candidate samples. Here, candidate samples refer to the unverified microbe-disease association pairs. If the rank of the test sample is higher than the given threshold, the proposed method is regarded as successful in inferring the tested microbe-disease pair. Similar to LOOCV, all observed microbe-disease association pairs are randomly divided into five groups in 5-fold CV. Each group is left out in turn to test model while the remaining groups are adopted as training samples. To weak the impact of the bias resulting from the progress of random division to experimental results, this progress is executed 100 times. It is worth noting that both the similarities of microbes and diseases need to be recalculated for each round in both LOOCV and 5-fold CV. For the sake of convenient observation, we draw the receiver-operating characteristics (ROC) curves by plotting true positive rate (TPR, sensitivity) against false positive rate (FPR, 1-specificity) based on different thresholds. Sensitivity represents the percentage of the true positive samples which are ranked higher than the given threshold in the whole positive samples. Specificity means the percentage of the negative samples with ranks lower than the given threshold in the whole negative samples. Area under ROC curve (AUC) is further calculated as a metric to measure the prediction capability of WMGHMDA. If the value of AUC is equal to 1, it means the model obtains perfect performance. If the value of AUC is equal to 0.5, it represents the performance of the model is random. As a result, WMGHMDA achieved an effective and reliable performance with average AUCs of 0.9288, 0.9068 ±0.0031 in the frameworks of LOOCV and 5-fold CV, respectively, as shown in Fig. 1.

For assessing the robustness of our method, we randomly select some known associations as unknown associations. The percentage varies from 10 to 30%. Global LOOCV is then implemented on the new known microbe-disease associations to evaluate the performance of our method. The results have been shown in Table 1, from which we can find that the AUCs are stable with the percentage increasing. It indicates that our method is slightly limited to the effect of sparse evidences.

**Table 1:** The robustness of WMGHMDA

Percentage	10%	20%	30%
AUC	0.9222	0.9161	0.9050

### Comparison with other methods

#### Do novo cross-validation

In order to further validate the sound prediction performance of the model, we compare WMGHMDA with some state-of-the-art computation methods, such as KATZHMDA [22], NTSHMDA [24], NGRHMDA [26], BiRWHMDA [41], LRLSHMDA [36], BDSILP [27] and PRWHMDA [42]. KATZHMDA is the first computation model to infer latent MDAs. It prioritizes candidate microbe-disease pairs according to their prediction scores obtained by calculating the numbers of walks with different lengths between microbe nodes and disease nodes. NTSHMDA is a global computational model that infers potential MDAs using optimized random walk with restart by introducing network topological similarity. NGRHMDA integrates two single recommendation algorithms, namely neighbor-based prediction model and graph-based prediction model, to calculate relationship probabilities of microbe-disease pairs and further prioritizes potential candidate microbes for diseases according to their probabilities. BiRWHMDA achieves possible microbe-disease association inference by exploring the CBGs through iteratively implementing random walk on the disease similarity networks and the microbe similarity network. LRLSHMDA is a semi-supervised computation model that uncovers potential MDAs by introducing Laplacian regularized least squares classifier. BDSILP is a network-based microbe prioritization model using label propagation. PRWHMDA finishes the inference of microbe-disease associations with extended RWR optimized by Particle Swarm Optimization. All of these methods perform great prediction performance. In the sake of fair comparison, these contrast approaches are implemented on the same database HMDAD, including 483 entries between 39 diseases and 292 microbes, as WMGHMDA. Both global LOOCV and 5-fold CV are adopted to measure the inference capabilities of experimental methods. As shown in Fig. 1, WMGHMDA outperforms baseline methods with an AUC of 0.9288 in LOOCV while the AUCs of KATZHMDA, NTSHMDA, NGRHMDA, BiRWHMDA, LRLSHMDA, BDSILP and PRWHMDA are 0.8382, 0.9070, 0.9111, 0.8964, 0.8909, 0.9131 and 0.9150, respectively. Similarly, in 5-fold CV, the performance of WMGHMDA is also superior to baseline methods (KATAHMDA 0.8301 ±0.0033; NTSHMDA 0.8896 ±0.0038; NGRHMDA 0.9023 ±0.0031; BiRWHMDA 0.8808 ±0.0029; LRLSHMDA 0.8794 ±0.0033, BDSILP 0.9030 ±0.0039; PRWHDMA 0.8870 ±0.0046) with average AUC of 0.9068 ±0.0031.

Furthermore, according to the result of global LOOCV, we obtain precision, recall and F1-score (See Additional file 1: Table S1) with different threshold *k*. We give the results of different methods with threshold *k* varying from 0 to 50 with a step value of 5. It can be clearly observed in Additional file 1: Table S1 that from the top-1 to -10 predictions, our model outperforms baseline methods in terms of these three evaluation metrics. For the predictions from top-10 to -50, our approach is comparable or even superior to baseline methods. It indicates that our method is effective in identifying candidate microbe for diseases. In addition, we can notice that the performances of some baseline methods (i.e., BiRWHMDA, NGRHMDA, KATZHMDA) are close to that of our approach. It could be explained that the difference of the prediction ability is possibly weakened by the highly skewed dataset where the number of unknown associations greatly exceeds the number of known associations in our database [43]. We believe that with the validation of more known evidences, the difference will become more evident.

#### Evaluate the performance of WMGHMDA in recovering known associations

**Fig. 2:**
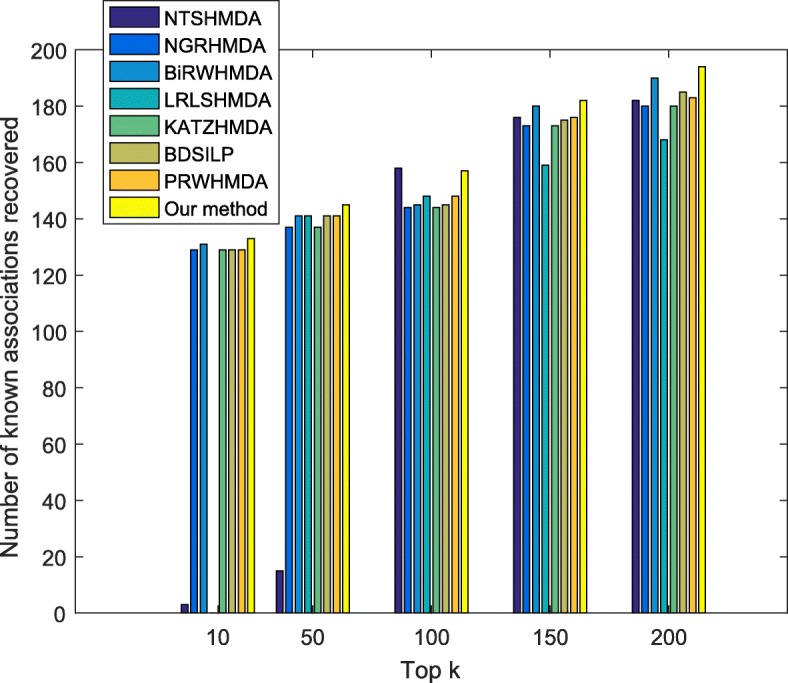
Performance comparisons between our method and baseline methods (NTSHMDA, NGRHMDA, BiRWHMDA, LRLSHMDA, KATZHMDA, BDSILP and PRWHMDA) in recovering known associations

In order to compare the ability of different methods in recovering a true association, we give the cumulative distribution of known associations recovered with top 10, 50, 100, 150 and 200 predictions, as shown in Fig. 2. Also, this result is obtained based on the result of global LOOCV. We can see in Fig. 2 that the number of known associations truly recovered by our method is more than those of baseline methods for all thresholds while our method is slightly inferior to NTSHMDA in top-100 prediction. Therefore, we can conclude that the developed model of WMGHMDA is effective and reliable, and, moreover, has comprehensively higher accuracy in inferring potential candidate microbes for diseases than state-of-the-art algorithms.

### Performance of prediction for new diseases

An important aspect to evaluate a novel model is the ability to make predictions for new diseases. New diseases refer to the diseases which have no any known experimentally verified associated microbes, but have prior features available for prediction. Due to the lack of known microbe associations, few existing computational methods are capable of inferring potential microbes for new diseases in the research field of MDAs. Therefore, one of our main contributions is that the proposed method is able to predict candidate microbes for new disease, which is achieved by taking prior information related to this disease and specific microbes into account. For the purpose of evaluation, we select the cumulative distribution of the ranks as a measure criterion to distinguish the prediction performance of different models for new diseases. This measure has been adopted for evaluation in multiple research fields of computational biology [44–47]. Considering that most of diseases included in HMDAD only have a small number of positive samples, and adopting them as test samples possibly results in the bias of evaluation, we choose three common diseases (Crohn’s disease, Liver cirrhosis, Type 1 diabetes) with more positive samples as test samples for more accurate evaluation.

**Fig. 3:**
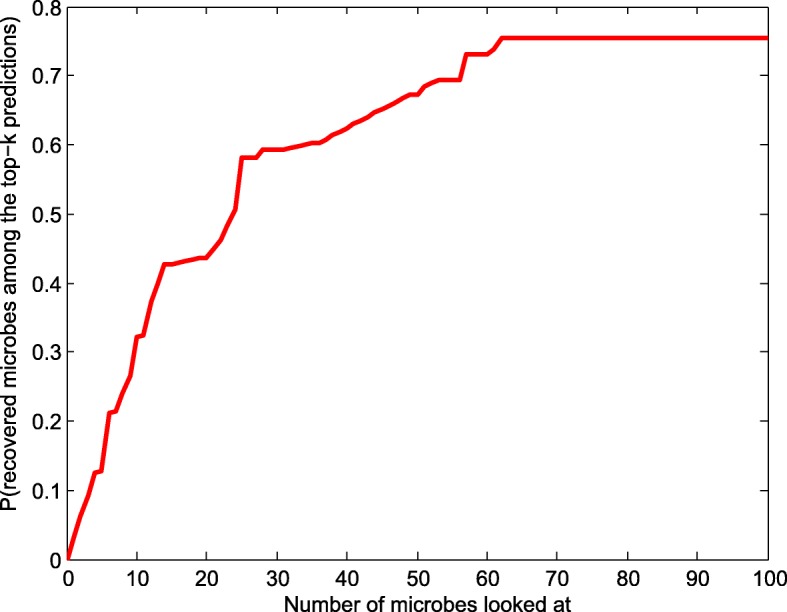
Performance of WMGHMDA in inferring candidate microbes for new diseases

For each of these diseases, we first artificially set all the known associations between microbes and test disease as unknown ones. And then different models are carried out on this test set to obtain the ranks of microbes which are experimentally verified to be associated with this test disease. After that, we can plot the average cumulative distribution of the ranks for three diseases, as shown in Fig. 3 where x-axis represents the top-*k* predicted microbes and y-axis denotes the probability of recovering an observed association in the top-*k* prediction. Note that all baseline methods are missing from the plot, as they cannot be applied for prediction for new diseases without any known associations. In Fig. 3, we can obviously observe that with the increase of number of microbes looked at, the percent of known true positive samples recovered by our method constantly increases. Especially, our model successfully recovers average 75.4% of known associations in the top-100 predictions. This performance can be explained because for any specific disease with known or unknown associations, in the framework of WMGHMDA, weighted meta-graph is capable of effectively capturing potential semantic associations between this disease and candidate microbes by combining prior biological knowledge associated with this disease and microbes. Thus, it can be concluded that our method is reliable and effective in predicting potential microbes for new diseases.

### The effects of parameters on WMGHMDA

**Fig. 4:**
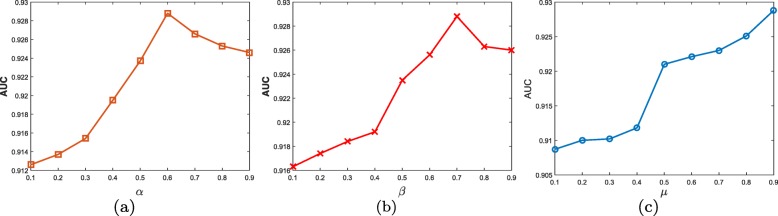
The impacts of parameters on the performance. (**a**) *α*, (**b**) *β*, (**c**) *μ*

In this section, we evaluate the impacts of parameters on the performance. *α* is weighted factor defined to weight the effects of the disease semantic similarity and the Gaussian kernel disease similarity to the integrated disease similarity. We set *α* from 0.1 to 0.9 with a step value of 0.1. *β* is a weighted factor used to control the contribution of the microbe functional similarity to the integrated microbe similarity. The setting of *β* is similar to *α* varying from 0.1 to 0.9. For the convenience of parameter tuning, one parameter is tested with the remaining parameters fixed. As shown in Fig. 4a, b, it can be obviously observed that the AUC first increases, and then decreases for both *α* and *β*. The best performance can be obtained when *α* and *β* are set as 0.6 and 0.7, respectively. It demonstrates that the values of *α* and *β* that are large or small are not good for the improvement of the prediction accuracy of our approach. *μ* is a weight factor controlling the contribution of weighted meta-graph to the prediction probability. Figure 4c shows that with *μ* increasing, the AUC always presents an upward trend, and we can acquire the best result when *μ* is set as 0.9. The result validates the effectiveness of weighted meta-graph.

### Case studies on asthma and inflammatory bowel disease

To further verify the prediction accuracy of WMGHMDA, we take asthma and Inflammatory bowel disease (IBD) as two case studies which are implemented on our model. All candidate microbes for asthma and IBD are prioritized according to their prediction scores. Here, the performance of WMGHMDA is evaluated by observing the number of confirmed candidate microbes ranked in the top of 10, 20, 50 for a specific disease. It is necessary to point out that for a given disease, if one microbe is associated with this disease, the corresponding genus of this microbe is also assumed to be related with this disease. Finally, 9, 19, 37 and 10, 20, 45 out of the top-10, 20, 50 candidate microbes could be manually validated based on previous literatures for asthma and IBD, respectively.

#### Asthma

Asthma is a common long-term inflammatory disease of the airway of the lungs [48]. An increasing number of statistics have shown that the microorganisms living in or on human hosts significantly get involved in the pathological mechanism of asthma. Nine out of top-10 microbes inferred to be associated with asthma obtain validation from different literatures. For example, Marri et al. [49] compared the changes of the microbiome of induced sputum from both asthmatic and nonasthmatic adults. As a result, they found that *Firmicutes* occurred in samples from nonasthmatic subjects with higher frequency. *Lachnospiraceae* was found to be significantly more prevalent in the sputum of asthma patients than in the sputum of the controls [50]. It was discovery that the abundance of *Enterobacteriaceae* of severe asthmatics was higher than that of non-severe asthmatics [51]. Yu et al. [52] showed that *Lactobacilli* were probiotic bacteria and had potential for preventing asthma. The abundances of *Staphylococcus* were presented to be larger in asthmatic children than those in healthy controls and asthmatics [53]. Vael et al., [54] investigated the relationship between the development of asthma and early intestinal colonization in the first 3 years of life, which, eventually, showed that the count of *Bacteroides fragillis* was significantly higher in children with a positive index compared to those without at 3 weeks. This result indirectly demonstrates that the change of *Bacteroides* is associated with asthma. Only *Clostridium difficile* has been not validated nowadays. Top-10 inferred candidate asthma-associated microbes are also listed in Table 2. Furthermore, 19, 37 out of top-20, 50 candidate microbes for asthma are confirmed manually by previously published literatures, as shown in Table 2.

**Table 2:** Prediction results of the top 50 asthma-associated microbes

Microbe	Evidence	Microbe	Evidence
Firmicutes	PMID:23265859	Treponema	Unconfirmed
Clostridium difficile	Unconfirmed	Porphyromonas gingivalis	PMID:20308298
Actinobacteria	PMID:28947029	Selenomonas	PMID:27093794
Clostridium coccoides	PMID:21477358	Escherichia coli	PMID:29161804
Lactobacillus	PMID:20592920	Clostridium leptum	PMID:29445257
Lachnospiraceae	PMID:27433177	Gammaproteobacteria	PMID:28947029
Staphylococcus	PMID:29445257	Fusobacterium nucleatum	[55]
Enterobacteriaceae	PMID:28947029	Alcaligenaceae	PMID:19407055
Bacteroides	PMID:18822123	Coriobacteriaceae	PMID:28947029
Veillonella	PMID:25329665	Erysipelotrichaceae	Unconfirmed
Clostridia	PMID:21477358	Methanobrevibacter smithii	Unconfirmed
Fusobacterium	[55]	Bacteroidaceae	PMID:28947029
Enterococcus	PMID:29788027	Verrucomicrobiaceae	Unconfirmed
Burkholderia	PMID:24451910	Dietzia maris	Unconfirmed
Streptococcus	PMID:17950502	Staphylococcus epidermidis	PMID: 29569134
Enterobacter aerogenes	PMID:24973962	Tropheryma whipplei	PMID:26647445
Enterobacter hormaechei	PMID:24973962	Acinetobacter	PMID:29447223
Klebsiella pneumoniae	PMID:24958709	Corynebacterium	PMID:29885665
Shigella dysenteriae	Unconfirmed	Oxalobacter formigenes	Unconfirmed
Propionibacterium	PMID:27433177	Desulfovibrio	Unconfirmed
Propionibacterium acnes	PMID:27433177	Lysobacter	Unconfirmed
Pseudomonas	PMID:13268970	Rickettsiales	Unconfirmed
Stenotrophomonas maltophilia	Unconfirmed	Streptococcus	PMID:17950502
Faecalibacterium prausnitzii	PMID:27253486	Xanthomonas	Unconfirmed
Actinomyces	PMID:23326024	Clostridium	PMID:26565810

The first column records top 1-25 associated microbes. The third column records top 26-50 associated microbes

#### Inflammatory bowel disease

Inflammatory bowel disease is a common group of inflammatory conditions of the colon and small intestine. Similar to asthma, WMGHMDA is also applied to infer possible related microorganisms for IBD. As a result, 10 of top-10 candidate IBD-related microbes could be confirmed by current researches. As an instance, it was demonstrated that the decreases of abundances of *Bacteroidete* and *Firmicute* were associated with the formation of IBD [56]. The abundance of *Clostridium coccoides* was discovered to be less represent in Crohn’s disease patients than healthy objects [57]. It was confirmed that there was an inversely association between the presence of *Heicobacter pylori* and IBD [58]. Azimirad et al. [59] indicated that there existed significant relationships between IBD and *Clostridiu difficile* and *staphylococcus*. Through observing the composition of salivary microbiota from 35 IBD patients, it was uncovered that *Haemophilus* largely contributed to dysbiosis observe in the salivary microbiota from IBD patients [60]. Ten out of top-10 predicted candidate microorganisms considered to be associated with IBD are also listed in Table 3, from which we can found that only *Enterobacteriaceae* has not been confirmed by current researches. In addition, 20 out of top-20 candidate, 45 out of top-50 candidate microbes for IBD are manually validated by current researches, as shown in Table 3. In addition, the network of the top-50 predicted associations for IBD and asthma obtained by our model is shown in Additional file 2: Figure S1. Obviously, it is observed that a microbe is possibly associated with one or more diseases. In a word, these two sets of case studies validate the powerful capability of our method in inferring new possible microbes for diseases again.

**Table 3:** Prediction results of the top 50 inflammatory bowel disease-associated microbes

Microbe	Evidence	Microbe	Evidence
Bacteroidetes	PMID:25307765	Clostridium	Azimirad et al.,2012
Firmicutes	PMID:25307765	Bacteroides ovatus	PMID:29454108
Prevotella	PMID: 25307765	Betaproteobacteria	Unconfirmed
Clostridium difficile	Azimirad et al.,2012	Clostridiales	PMID:29965986
Helicobacter pylori	PMID:22221289	Klebsiella	PMID:29573336
Clostridium coccoides	PMID:19235886	Bifidobacterium	PMID:24478468
Staphylococcus	Azimirad et al.,2012	Gammaproteobacteria	PMID:29385143
Lactobacillus	PMID:26340825	Porphyromonadaceae	PMID:29573237
Haemophilus	PMID:24013298	Collinsella aerofaciens	PMID:26848182
Enterobacteriaceae	PMID:24629344	Propionibacterium	PMID:26640113
Staphylococcus	Azimirad et al.,2012	Propionibacterium acnes	PMID:26640113
Veillonella	PMID:28842640	Alistipes	PMID:28877044
Bacteroides	PMID:25307765	Parabacteroides	PMID:25307765
Clostridia	PMID:25307765	Prevotellaceae	PMID:29514953
Bacteroides vulgatus	PMID:29454108	Veillonellaceae	PMID: 28842640
Bacteroides uniformis	PMID:26789999	Fusobacteriaceae	PMID:24629344
Bacteroidaceae	PMID:17897884	Shigella	PMID:29485143
Faecalibacterium prausnitzii	PMID:24799893	Enterobacter aerogenes	Unconfirmed
Streptococcus	PMID:23679203	Enterobacter hormaechei	Unconfirmed
Clostridium leptum	PMID:28099495	Klebsiella pneumoniae	PMID:29573336
Enterococcus	PMID:24629344	Coxiellaceae	Unconfirmed
Escherichia coli	PMID:29573336	Bacteroidales	PMID:24629344
Stenotrophomonas maltophilia	Uncofirmed	Enterococcus faecium	PMID:29135456
Fusobacterium	PMID:25307765	Erysipelotrichales	PMID:29965986
Burkholderia	PMID:24325678	Bacilli	PMID:29049404

The first column records top 1-25 associated microbes. The third column records top 26-50 associated microbes

## Discussion

Evidences showed that the microbes living in or on human body significantly contributed to the induction progress of an extensive varieties of complex human diseases, including formation, development and progression. Inferring latent candidate microbes for diseases can not only provide significant insights into the understanding of the pathological mechanism of complex diseases, but also promote disease prevention, diagnosis and treatment, as well as drug development. In this study, we proposed a novel Weighted Meta-Graph based computational method of WMGHMDA to predict potential microbe-disease associations based on HIN. The experimental results indicated that our method achieved a desired improvement compared to some state-of-the-art methods. Our method made full use of multiple prior biological knowledge.

In particular, we integrated disease semantic similarity and microbe functional similarity to complement and improve the disease similarity and microbe similarity, respectively. This prior information is essential for the predictions for new diseases. The prediction experiments for three common complex diseases indicated that prior information was helpful for making predictions for new diseases. In fact, the introduction of prior information also alleviates the problem that previous computational methods tend to "recommend" well-investigated candidate microbes or diseases. In this study, we introduced meta-graph to solve the problem of inferring potential associations between diseases and microbes with the consideration of its power in capturing complex semantics in HIN. Further, inspired by the fact that there are prolific subtle semantics hidden in HIN, we generalized unweighted meta-graph to weighted meta-graph to more accurately capture them. In addition, weighted meta-graphs with diverse patterns are likely to lead to differentiated contributions to a microbe-disease pair. Thus, to identify such differences and enhance the prediction accuracy, we further introduced bias rating to describe the distinct contributions of different weighted meta-graph. The comprehensive experimental results indicated that the introduction of weighted meta-graph can improve prediction performance.

The reliable performance of WMGHMDA results from several major factors as follows: to begin with, the observed experimentally validated human MDAs are reliable. In addition, the introduction of multiple prior biological information about diseases and microbes improves the completeness of similarity for diseases and microbes, which potentially enhances the prediction capability of our method. Last but not least, a crucial advantage of WMGHMDA is that it achieves potential MDAs inference based on weighted meta-graph. On the one hand, compared with unweighted meta-graph, weighted meta-graph has stronger ability to capture potential subtle semantic associations between seed diseases and target microbes. On the other hand, for a microbe-disease pair, the bias contributions of different weighted meta-graphs to it are considered in this paper. Weighted meta-graph with higher bias rating is assigned greater weight value when probability score is calculated, which also promotes the improvement of the prediction performance.

Although the performance of WMGHMDA is desirable, several aspects are still expected to be further improved in future studies. Initially, the available of known MDAs is still not enough to insure more desirable prediction performance, which, however, could be addressed by adding manually more known microbe-disease association to database. Furthermore, it is greatly easy for the proposed model to suffer from decrease of accuracy owing to the high rate of false positive and false negative samples in the microbe-disease association database. Additionally, our method cannot be applied to make predictions for all new diseases. It is because for a new disease without any known evidences and DAG information, it fails to obtain its similarity between other diseases and it that is essential for new disease prediction. But this limitation could be overcome by incorporating more prior information or developing other effective similarity calculation method.

Finally, it is anticipated that the prediction accuracy of microbe-disease association could be improved through two aspects. On one hand, more prior biological knowledge could be introduced, such as microbe sequence similarity, disease gene-based similarity network and disease symptom similarity network. Compared with the study of disease similarity, the attention paid on the study of microbe similarity are relatively poor. It is an alternative way to adopt the combination of CRISPR-Cas9 with functional enrichment to measure microbe sequence similarity by first mapping genetic interaction network based on microbial sequencing data and then detecting similar features on the network. On the other hand, computational approaches have been fully developed in other computational biology fields, such as microRNA-disease association prediction. Inspired by the advanced computational methods in these fields, we expect to develop more effective computational model.

## Conclusion

Identifying potential microbe-disease associations is a primary step towards understanding the pathological mechanism of human diseases. In this study, we proposed a Weighted Meta-Graph-based computational method for disease-microbe association prediction. We compared our method with several state-of-the-art methods based on database HMDAD. According to the experimental results, it indicated that our method performed better than baseline methods. Also, we applied our method to make predictions for three common human diseases to validate its effectiveness for new diseases. As a result, our method achieved a desired prediction performance. In addition, in case studies, most of the inferred candidate microbes could be validated by previous reports. Therefore, we believe that the proposed method has potential to investigate the underlying pathological mechanism of human diseases.

## Methods

### Human microbe-disease associations

The known experimentally validated human microbe-disease association data were retrieved from Human Microbe-Disease Association Database (HMDAD, http://www.cuilab.cn/hmdad.) which contains 483 distinct experimentally validated microbe-disease entries involving 39 diseases and 292 microbes [21]. For the sake of convenience, we construct an adjacency matrix $A \in {R^{n_{d} \times n_{m}}}\phantom {\dot {i}\!}$ to represent the known human microbe-disease associations, where *n*_*d*_ denotes the number of diseases while *n*_*m*_ the number of microbes. If there exists experimentally confirmed association between disease *d*_*i*_ and microbe *m*_*j*_, then *A*_*ij*_ equals to 1, otherwise 0.

### Microbe functional similarity

In this paper, we calculate microbe functional similarity based on the method proposed by Kamneva et al. [61]. In order to accurately calculate the functional similarity for a given pair of microbes, we first need to obtain a protein-protein functional association network where the nodes represent gene families encoded by either of the genomes and the links represent gene neighbor score values based on STRING database (https://string-db.org.). Gene families are labeled to denote if a protein from a given gene family is present in genome A, genome B, or both, which produces 3 types of gene families. There exist 6 types of undirected edges (both to A, both to B, both to both, A to A, A to B and B to B) in such a network. We define the microbe functional similarity between two microbes as a fraction of edges which cross organismal boundaries (i.e. A to B) among all the edges connecting gene families encoded exclusively in one of the genomes (i.e. A to B, A to A and B to B). A simple example is shown in Additional file 3: Figure S2. The similarity scores are transformed into a *n*_*m*_×*n*_*m*_ microbe functional similarity matrix *FS* where *FS*(*m*_*i*_,*m*_*j*_) represents the similarity between microbe *m*_*i*_ and microbe *m*_*j*_.

### Disease semantic similarity

Mesh (Medical Subject Headings) database (http://www.ncbi.nlm.nih.gov/.) includes a plenty of descriptors about diseases, based on which a Directed Acyclic Graph (DAG) can be constructed to describe a disease [62]. The DAG of disease *D* is composed of not only its ancestor nodes and *D* itself but also the directed edges from patient nodes to child nodes. Based on the DAG, we can define the contribution value of disease *d* in *DAG*(*D*) to the semantic value of disease *D* as follows:

1 $$ \left\{\begin{array}{l} S{V_{D}}(d) = 1,\;\;\;\;\;\;\;\;\;\;\;\;\;\;\;\;\;\;\;if\;\;d = D,\\ S{V_{D}}(d) = \max \left\{{\Delta * S{V_{D}}({d^{'}})|{d^{'}} \in children\;of\;d} \right\},\\\;\;\;\;\;\;\;\;\;\;\;\;\;\;\;\;\;\;\;\;\;\;\;\;\;\;\;\;\;\;\;\;\;\;\;\;\;\;\;if\;d \ne D, \end{array} \right.  $$

where *Δ* represents the semantic contribution decay factor utilized to limit the effects of diseases with different distances to disease *D* (According to Wang et al. [63], we set *Δ* as 0.5). Generally, the larger the distance of disease *D* to its ancestor disease is, the less its contribution to the semantic value of disease *D* is. The semantic value of disease *D* can be defined as follows:

2 $$ SV(D) = {\sum\nolimits}_{d \in T(D)} {S{V_{D}}(d)},  $$

where *T*(*D*) represents all the ancestor diseases of disease *D* and *D* itself. Based on the assumption that the larger the shared part of the DAGs of two diseases is, the greater their similar score is, the semantic similarity value between disease *d*_*i*_ and disease *d*_*j*_ could be defined as follows:

3 $$ SS({d_{i}},{d_{j}}) = \frac{{{\sum\nolimits}_{t \in T({d_{i}}) \cap T({d_{j}})} {(S{V_{d_{i}}}(t) + S{V_{d_{j}}}(t))} }}{{SV({d_{i}}) + SV({d_{j}})}}.  $$

### Gaussian interaction profile kernel similarity for microbes

Based on the assumption that microbes with similar functions tend to present interaction or non-interaction with similar diseases [21], we construct microbe similarity network and disease similarity network via known experimentally confirmed human microbe-disease interaction relationships using Gaussian kernel interaction profile, respectively. For a specific microbe *m*_*i*_, the corresponding interaction profile could be denoted as *IP*(*m*_*i*_), which describes the interaction relationships between microbe *m*_*i*_ and all considered diseases, i.e., if a disease is confirmed experimentally to be associated with *m*_*i*_, the corresponding value of *IP*(*m*_*i*_) equals to 1, otherwise 0. According to the interaction profiles, the Gaussian kernel microbe similarity *GM* can be calculated and defined as follows [29]:

4 $$ GM(m_{i},m_{j}) = \exp \left(- {\lambda_{m}}{\left\| {IP(m_{i}) - IP(m_{j})} \right\|^{2}}\right),  $$

5 $$ {\lambda_{m}} = \lambda_{m}^{\prime}/\left(\frac{1}{{{n_{m}}}}\sum\limits_{i = 1}^{{n_{m}}} {{{\left\| {IP(m_{i})} \right\|}^{2}}}\right),  $$

where *λ*_*m*_ represents the normalized kernel bandwidth, and can be updated by another normalized bandwidth $\lambda _{m}^{\prime }$. For convenience, we set $\lambda _{m}^{\prime } = 1$ according to previous relevant research [63]. *n*_*m*_ is the number of microbes. *GM*(*i*,*j*) at the *i*^*th*^ row and *j*^*th*^ column denotes the similarity between microbe *m*_*i*_ and *m*_*j*_.

### Gaussian interaction profile kernel similarity for diseases

Similarly, the Gaussian kernel disease similarity *GD* can be computed as follows:

6 $$ GD(d_{i},d_{j}) = \exp \left(- {\lambda_{d}}{\left\| {IP(d_{i}) - IP(d_{j})} \right\|^{2}}\right),  $$

7 $$ {\lambda_{d}} = \lambda_{d}^{\prime}/\left(\frac{1}{{{n_{d}}}}\sum\limits_{i = 1}^{{n_{d}}} {{{\left\| {IP(d_{i})} \right\|}^{2}}}\right),  $$

where $\lambda _{d}^{\prime }$ is also set to 1 and *n*_*d*_ is the number of diseases. *GD*(*i*,*j*) at the *i*^*th*^ row and *j*^*th*^ column implies the similarity between disease *d*_*i*_ and *d*_*j*_.

### Integrated similarity for diseases

In order to complement and improve disease similarity, we construct a new similarity network for diseases by combining multiple disease similarity networks calculated from different perspective, namely the disease semantic similarity and the Gaussian kernel disease similarity, as is mentioned above. Specifically, the integrated disease similarity can be defined as follows:

8 $$ DS({d_{i}},{d_{j}}) = \left\{ \begin{array}{l} {{\alpha}SS({d_{i}},{d_{j}}) + (1-\alpha)GD({d_{i}},{d_{j}})},\\ if\;{d_{i}}\;and \;{d_{j}}\;has\;semantic\;similarity,\\ GD({d_{i}},{d_{j}}),\;\;\;\;\;\;\;\;\;\;\;\;\;\;\;otherwise, \end{array} \right.  $$

where *α* is weight factor defined to limit the effects of the disease semantic similarity and the Gaussian kernel disease similarity to the combined disease similarity. The values of these parameters are determined by the experimental results.

### Integrated similarity for microbes

Similarly, a new similarity network for microbes is constructed by integrating microbe functional similarity and Gaussian kernel microbe similarity. Formally, the integrated microbe similarity can be calculated as follows:

9 $$ MS = \beta FS + (1 - \beta)GM  $$

where *β* is a weight factor used to weight the impacts of the Gaussian kernel microbe similarity and the microbe functional similarity to the final combined microbe similarity.

### Construction of heterogeneous information network

Based on the calculated similarities for diseases and microbes, we can further construct disease similarity network and microbe similarity network, based on which a HIN can be constructed through known experimentally validated MDAs. As for microbe similarity network, $M = \{ {m_{1}},{m_{2}}, \ldots,{m_{{n_{m}}}}\} \phantom {\dot {i}\!}$ implies the node set of microbes and the edge weights denote the similarities between microbes. Similarly, as for the disease network, $D = \{ {d_{1}},{d_{2}}, \ldots,{d_{{n_{d}}}}\} \phantom {\dot {i}\!}$ denotes the node set of diseases and the edge weights represent the similarities between diseases. In addition, a bipartite network is also constructed in the HIN with the node set consisting of microbe and disease nodes and the edge weights representing the absence or presence of relationships between diseases and microbes, i.e., if there is an edge between *d*_*i*_ and *m*_*j*_, it implies that *d*_*i*_ is experimentally confirmed to be related with *m*_*j*_ and the corresponding edge weight equals to 1, otherwise 0.

### WMGHMDA

**Fig. 5:**
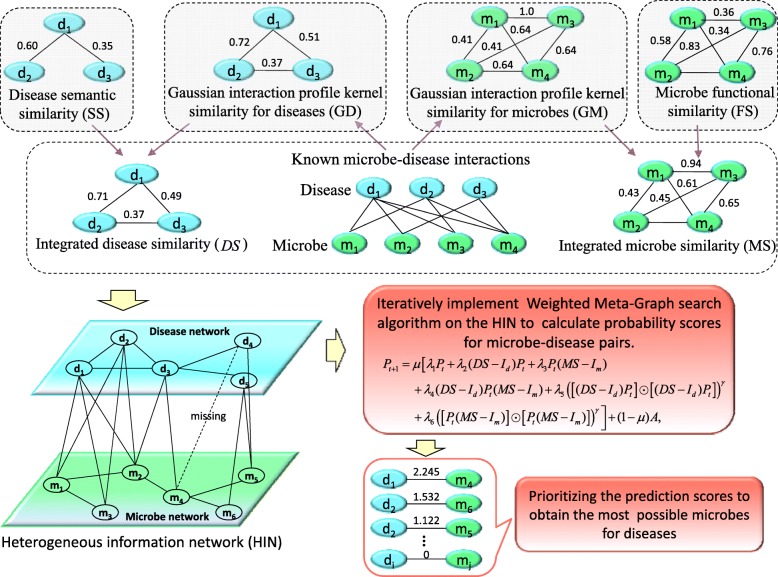
The flowchart of WMGHMDA model to predict potential human microbe-disease associations. The first step is constructing a heterogeneous network by connecting the microbe similarity network where the microbe similarity is obtained by combining the Gaussian kernel microbe similarity with the microbe functional similarity, the disease similarity network where the disease similarity is obtained by combining the Gaussian kernel disease similarity with the disease semantic similarity, and the known microbe-disease association network. The second step is iteratively executing Weighted Meta-Graph search algorithm on the heterogeneous network to calculate the scores of the microbe-disease pairs. Finally, prioritizing candidate microbes for diseases according to their scores

In this work, we developed a novel Weighted Meta-Graph based computational framework for predicting microbe-disease associations (WMGHMDA). The flowchart of WMGHMDA is shown in Fig. 5, Firstly, to improve the completeness of similarity, we obtain the integrated disease similarity by combining disease semantic similarity with Gaussian kernel disease similarity, and the integrated microbe similarity by combining microbe functional similarity with Gaussian kernel microbe similarity, based on which a HIN is constructed via known microbe-disease interaction network. Secondly, we design a Weighted Meta-Graph search algorithm and implement it on the HIN to calculate the probability score for each microbe-disease pair. Finally, for a disease, all candidate microbes are prioritized according to their probability scores.

#### Meta-graph

The concept of meta-graph has been developed to capture more complex semantics in HIN that meta-path cannot[64]. Since each particular meta-graph represents an essential semantic unit between a source node and a target node in HIN, meta-graph has been widely applied in representation learning and recommendation system [65–68]. Inspired by this, we extend meta-graph to solve the problem of uncovering missing MDAs based on HIN. Here, we focus on the concepts related to our paper. Specially, we define the meta-graph in heterogeneous biological network for prediction.

**Fig. 6:**
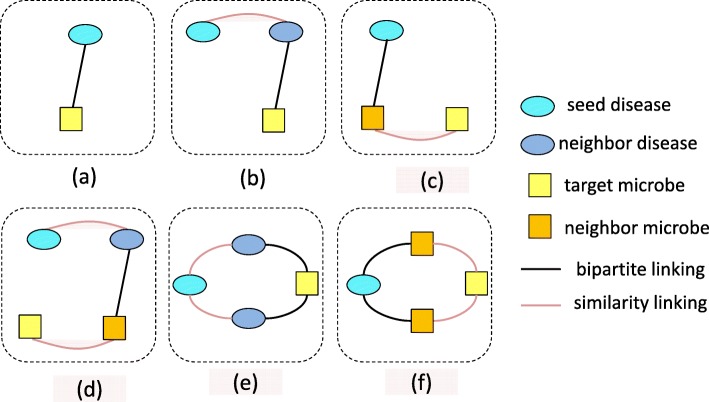
Examples of weighted meta-graphs used for microbe-disease association prediction. Red line represents the similarity linking between diseases that weights similarity degree; Black line is bipartite linking that denotes whether a disease is associated with a microbe or not, i.e., if a disease is confirmed to be related to a microbe, the weight value of corresponding edge equals to 1, otherwise 0. The numbers of the given nodes directly connected to the bipartite edge are 2, 1, 1, 0, 1, 1 from (**a**) to (**f**), respectively

Meta-graph is the subset of HIN schema. Formally, meta-graph could be defined as sub-graph *G*_*s*_=(*V*,*E*), where *V*={*d*_*i*_|*i*=1,2,…,*n*_*d*_}∪{*m*_*j*_|*j*=1,2,…,*n*_*m*_} represents the set of nodes including diseases and microbes, and *E*={(*v*_*i*_,*v*_*j*_)|*i*,*j*=1,2,…,*n*, *n*∈(*n*_*d*_∪*n*_*m*_)} implies the set of edges including inter-layer relationship connections in the bipartite network and intra-layer similarity connections in both of disease similarity network and microbe similarity network. A meta-path is a special case of a meta-graph. Here, we call it meta-graph uniformly. Figure 6 displays six types of meta-graphs which depict possible semantic relations between a seed disease node and a target microbe node. Here we regard the products of the weight values of all edges existing in a meta-graph as its contribution value to the prediction probability of the microbe-disease association pair. For example, for the given disease *d*_*i*_ and microbe *m*_*j*_, the contribution value of a meta-graph to the probability score of the pair could be defined and calculated as follows if there exists no observed relationship between them (assuming that the meta-graph is linear and includes less than three intermediate nodes):

10 $$ P({d_{i}},{m_{j}}) = \sum\limits_{k = 1}^{{n_{d}}} {\sum\limits_{t = 1}^{{n_{m}}} {DS({d_{i}},{d_{k}})} }A({d_{k}},{m_{t}})MS({m_{t}},{m_{j}}).  $$

#### Weighted meta-graph based prediction model

In order to more accurately capture potential subtle semantics between disease nodes and microbe nodes, here we generalize common unweighted meta-graph to weighted meta-graph. In the weighted meta-graph, the weight values of intra-layer edges represent the similarities between diseases or microbes, and the weight values of inter-layer edges denote the possibilities of existing associations between diseases and microbes, i.e., if a disease is experimentally verified to be related to a microbe, the weight value of corresponding bipartite edge equals to 1, otherwise 0. Empirically, as the number of the edges and the intermediate nodes of a meta-graph increases, the importance of the meta-graph also gradually decreases. Thus, based on this, we only adopt six types of weighted meta-graph patterns (as shown in Fig. 6), with the number of edges less than five or the number of intermediate nodes less than three, to identify latent MDAs in HIN. They include weighted meta-graphs with single-path (such as Fig. 6a,b,c,d) and weighted meta-graphs with dual-path (such as Fig. 6e,f). With the above-mentioned definition of the contribution of meta-graph, for these six different types of weighted meta-graphs, the corresponding contribution values could be described as follows according to formula (10), respectively:

11 $$ {P_{a}}({d_{i}},{m_{j}}) = A({d_{i}},{m_{j}}),  $$

12 $$ {P_{b}}({d_{i}},{m_{j}}) = \sum\limits_{k = 1,\;k \ne i}^{{n_{d}}} {DS ({d_{i}},{d_{k}})A({d_{k}},{m_{j}})},  $$

13 $$ {P_{c}}({d_{i}},{m_{j}}) = \sum\limits_{t = 1,t \ne j}^{{n_{m}}} {A({d_{i}},{m_{t}})MS({m_{t}},{m_{j}})},  $$

14 $$ \begin{aligned} {P_{d}}({d_{i}},{m_{j}})=&\sum\limits_{k = 1,k \ne i}^{{n_{d}}}{\sum\limits_{t = 1,t \ne j}^{{n_{m}}}}\\ &\left[{DS({d_{i}},{d_{k}})} A({d_{k}},{m_{t}})MS({m_{t}},{m_{j}})\right], \end{aligned}  $$

15 $$ \begin{aligned} {P_{e}}({d_{i}},{m_{j}}) = &\sum\limits_{k = 1,k \ne i}^{{n_{d}}} {\sum\limits_{t = 1,t \ne i,t \ne k}^{{n_{d}}}}\\ &{\left[DS ({d_{i}},{d_{k}})A({d_{k}},{m_{j}})DS({d_{i}},{d_{t}})A({d_{t}},{m_{j}})\right]}, \end{aligned}  $$

16 $$ \begin{aligned} {P_{f}}({d_{i}},{m_{j}}) = &\sum\limits_{k = 1,k \ne j}^{{n_{m}}} {\sum\limits_{t = 1,t \ne j,t \ne k}^{{n_{m}}} }\\ &{\left[{A({d_{i}},{m_{k}})MS({m_{k}},{m_{j}})A({d_{i}},{m_{t}})MS({m_{t}},{m_{j}})}\right] }. \end{aligned}  $$

However, weighted meta-graphs with different structure characteristics could actually yield bias contributions to a microbe-disease pair. Here, for identifying this bias, we introduce bias rating to describe the differentiated contributions of different weighted meta-graphs. The main differences between weighted meta-graphs depend on the number of the given nodes. Here, the given node refers to the node that is directly connected to the bipartite edge and could be a seed disease node as well as a target microbe node. Specifically, as shown in Fig. 6, different numbers of the given nodes are included in these six kinds of weighted meta-graphes. For example, the numbers of the given nodes for Fig. 6a-f are 2, 1, 1, 0, 1, 1, respectively. Based on the assumption that meta-graph with more given nodes has greater contribution potential, it indicates that compared with the other weighted meta-graphs, Fig. 6a possibly has more potential to contribute useful information to an association pair while Fig. 6d could contribute the least useful information. Note that although Fig. 6b,c have the same numbers of the given nodes as Fig. 6e,f, the later may play more important role in predicting candidate microbes for diseases. It can be explained that both Fig. 6e and f are dural-path weighted meta-graphs, which implies that a seed disease node has more semantic paths simultaneously connecting it to a target microbe node in such meta-graph. In other words, such weighted meta-graph can hide more prolific semantic information, implying more contribution potential. Therefore, depending on the potential of contribution, we assign different bias ratings for different weighted meta-graphs. The greater the potential of contribution is, the higher the bias rating is.

According to the definition of weighted meta-graph, it can be found that a given microbe-disease association pair can be hidden in multiple varieties of weighted meta-graphs in HIN. Based on the assumption that more weighted meta-graphs are determined to be related to a microbe-disease pair, the pair is more likely to have association, the accumulating contribution values of all weighted meta-graphs connecting a seed disease with a target microbe can be served as their final prediction probability. Mathematically, for specific disease *d*_*i*_ and microbe *m*_*j*_, after implementing Weighted Meta-Graph search algorithm on the HIN to traverse all relevant weighted meta-graphs, the prediction score $\overline P$ could be defined and calculated by summing up the contribution values of these weighted meta-graphs as follows:

17 $$ \overline P ({d_{i}},{m_{j}}) = \sum\limits_{l = 1}^{N} {\sum\limits_{r = 1}^{M} {\lambda_{l}^{r}P_{l}^{r}({d_{i}},{m_{j}})} }  $$

where $P_{l}^{r}({d_{i}},{m_{j}})$ denotes the contribution value of the *r*^*th*^ meta-graph belonging to the *l*^*th*^ type of weighted meta-graph to the pair (*d*_*i*_,*m*_*j*_),*N* (*N*=6) represents the category number of weighted meta-graph, and *M* denotes the number of the weighted meta-graph included in a specific weighted meta-graph pattern. *λ*(*λ* ∈[0,1]) is bias rating applied to distinguish the contributions of different types of weighted meta-graphs to the final predicted probability $\overline P $. It is noteworthy that all weighted meta-graphs in the same category are considered to present identical bias ratings on a microbe-disease pair. We iteratively implement the above search progress based on Weighted Meta-Graph search algorithm until the prediction probability matrix *P*_*t*_ converges and describe the iteration formula with matrix formation as follows:

18 $$ \begin{aligned} {P_{t + 1}} = &\mu \left[{{\lambda_{1}}{P_{t}} + {\lambda_{2}}(DS - {I_{d}}){P_{t}} + {\lambda_{3}}{P_{t}}(MS - {I_{m}})} \right.\\ &\left.{+ {\lambda_{4}}(DS - {I_{d}}){P_{t}}(MS - {I_{m}})} \right.\\ &\left.{+ {\lambda_{5}}{{\left({\left[ {(DS - {I_{d}}){P_{t}}} \right] \odot \left[ {(DS - {I_{d}}){P_{t}}} \right]} \right)}^{\gamma} }}\right.\\ &\left.{+ {\lambda_{6}}{{\left({\left[ {{P_{t}}(MS - {I_{m}})} \right] \odot \left[ {{P_{t}}(MS - {I_{m}})} \right]} \right)}^{\gamma}} }\right] \\ &+ (1 - \mu)A, \end{aligned}  $$

where *I*_*d*_ and *I*_*m*_ represent unit matrices with the sizes of *n*_*d*_ and *n*_*m*_, respectively, and *λ* is bias rating (According to the experimental results, the best performance is obtained when *λ*_1_=0.35, *λ*_2_=0.1, *λ*_3_=0.1, *λ*_4_=0.05, *λ*_5_=0.2 *and* *λ*_6_=0.2.). The element of probability matrix *P*_*t*_ at the *i*^*th*^ row and *j*^*th*^ column means the probability score of association between disease *d*_*i*_ and microbe *m*_*j*_ at step *k*. ⊙ denotes Hadamard product, *γ* is a decay coefficient used to control the contributions of dural-path weighted meta-graphs (Here, we set *γ* as 0.1.), and *μ*∈(0,1) is a decay factor similar to the restart probability in the random walk with restart. The initial values of probability matrix *P*_*t*_ is defined as the normalized adjacent matrix *A*. According to Wang et al. [69], it assures that formula (18) will converge if *DS* and *MS* are properly normalized using Eqs. (19) and (20), respectively.

19 $$ DS ({d_{i}},{d_{j}}) = \frac{{DS ({d_{i}},{d_{j}})}}{{\sqrt {{\sum\nolimits}_{l = 1}^{{n_{d}}} {DS ({d_{i}},{d_{l}})}} \cdot \sqrt {{\sum\nolimits}_{l = 1}^{{n_{d}}} {DS ({d_{l}},{d_{j}})}} }},  $$

20 $$ MS({m_{i}},{m_{j}}) = \frac{{MS({m_{i}},{m_{j}})}}{{\sqrt {{\sum\nolimits}_{l = 1}^{{n_{m}}} {MS({m_{i}},{m_{l}})}} \cdot \sqrt {{\sum\nolimits}_{l = 1}^{{n_{m}}} {MS({m_{l}},{m_{j}})}} }}.  $$

After some steps, the prediction probability *P*_*t*_ is steady, according to which all candidate microbes for each disease could be prioritized. The top microbes are considered as the most possible microbes associated with the given disease.

The main time complexity of the algorithm is from the search of meta-graph and the corresponding calculation of contribution values. Given that the numbers of disease and microbe are *n*_*d*_ and *n*_*m*_, respectively, for six types of weighted meta-graphs (i.e. Fig. 6a-f), this process takes *O*(*n*_*d*_*n*_*m*_),*O*(*n*_*d*_^2^*n*_*m*_),*O*(*n*_*d*_*n*_*m*_^2^),*O*(*n*_*d*_^2^*n*_*m*_^2^),*O*(*n*_*d*_^3^*n*_*m*_) and *O*(*n*_*d*_*n*_*m*_^3^) in the worst case scenario by considering each disease node as seed node while each microbe node as target node, respectively. Therefore, the time complexity of the algorithm is *O*(*n*_*d*_*n*_*m*_(*n*_*d*_+*n*_*m*_)^2^). Our algorithm is implemented on Matlab R2016a.

### Implement wMGHMDA on new diseases

**Fig. 7:**
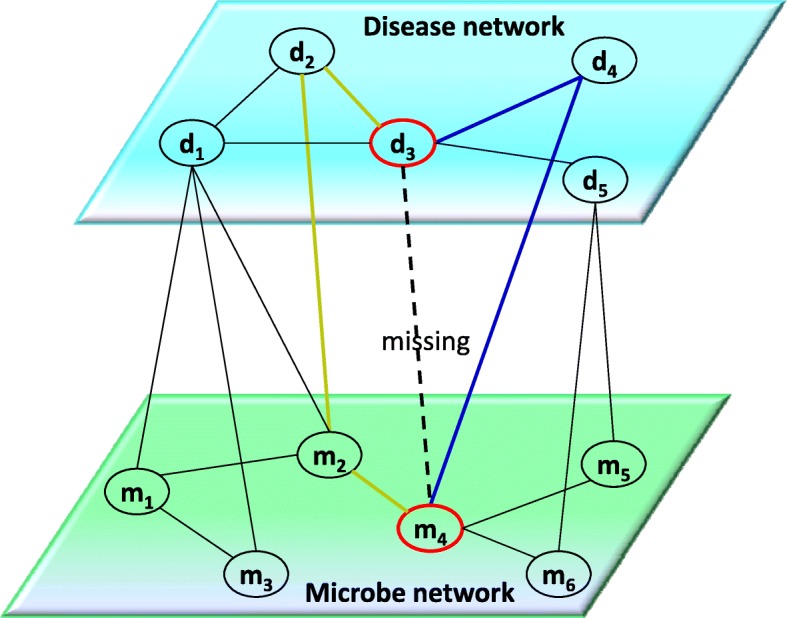
The heterogeneous information network. Two types of weighted meta-graphs (i.e. Fig. 6b,d) can be utilized to obtain the probability score of interaction between new disease node *d*_3_ and candidate microbe node *m*_4_, such as weighted meta-graphs consisting of *d*_3_,*d*_4_,*m*_4_ and *d*_3_,*d*_2_,*m*_2_,*m*_4_, respectively. *d*_3_ represents unlabeled disease; *d*_1_,*d*_2_,*d*_4_ and *d*_5_ denote labeled disease; *m*_1_,*m*_2_,*m*_3_,*m*_4_,*m*_5_ and *m*_6_ represent labeled microbes; Solid lines and dotted line in the bipartite network means known and unknown bipartite linkings, respectively

For new diseases which lack known associated microbes in the database but have other features available for prediction, few previous computational methods could be applied to make predictions. We implement WMGHMDA on new diseases for exploring potential microbes. One of the advantages of weighted meta-graph is that it is able to effectively capture the hidden semantic associations for microbe-disease pairs on the HIN. WMGHMDA embeds weighted meta-graph with multiple prior features related to diseases and microbes, such as disease semantic similarity and microbe functional similarity, which provides a possibility to bridge a new disease node with microbe node in HIN. Therefore, for new diseases, although there are no evidences to confirm their associations between microbes and them, WMGHMDA can still be applied to make predictions. An example is shown in Fig. 7.

Given a specific unlabelled disease *d*_3_, for each of association pairs between *d*_3_ and *m*_*j*_, it is easy to seek relevant weighted meta-graphs hidden in the HIN. For example, we can find two types of weighted meta-graphs related to association pair *d*_3_−*m*_4_, such as weighted meta-graphs consisting of *d*_3_,*d*_4_,*m*_4_ and *d*_3_,*d*_2_,*m*_2_,*m*_4_, respectively. If there is a higher similarity between the node *d*_3_ and a labeled disease node *d*_*i*_ (i.e., *d*_2_) or between the specific microbe node *m*_*j*_ (i.e., *m*_4_) and a labeled microbe node *m*_*k*_ (i.e., *m*_2_), it means that disease *d*_3_ is associated with microbe *m*_*j*_ with greater probability. After the Weighted Meta-Graph search algorithm is implemented, each microbe in the HIN will obtain a probability score denoting the possibility of being associated with new disease *d*_3_. The greater score indicates closer interaction between the microbes and *d*_3_. The probability scores can be calculated according to formula (17).

## Supplementary information

**Additional file 1** **Table S1**. Performance comparisons between our method and baseline methods (NTSHMDA, nGRHMDA, biRWHMDA, lRLSHMDA, kATZHMDA, bDSILP and pRWHMDA) in terms of precision, recall, and f1-score, respectively.

**Additional file 2** **Figure S1**. Network of the top-50 predicted associations for iBD and asthma obtained by our method. ellipses with Orange and circles represent diseases and microbes, respectively. the blue lines and red lines denote the associations of predicted microbes with iBD and asthma, respectively.

**Additional file 3** **Figure S2**. A simple example of how microbe functional similarity is calculated.

## Data Availability

The datasets supporting the conclusions of this article are included within the article and its Additional files. The code used in the current study is available at https://github.com/yahuilong/WMGHMDA.

## Abbreviations

LOOCV: Leave-one-out cross validation
AUC: Area under ROC curve
DAG: Directed acyclic graph
HIN: Heterogeneous information network
HMDAD: Human microbe-disease association database
RWR: Random walk with restart
MDAs: Microbe-disease associations
WMGHMDA: Weighted meta-graph-based method for human microbe disease association prediction
