# Supplementary figures and images for: WMGHMDA: a novel weighted meta-graph-based model for predicting human microbe-disease association on heterogeneous information network
Source: BMC Bioinformatics. 2019 Nov 1;20:541. doi: 10.1186/s12859-019-3066-0 (PMC6824056; doi:10.1186/s12859-019-3066-0)

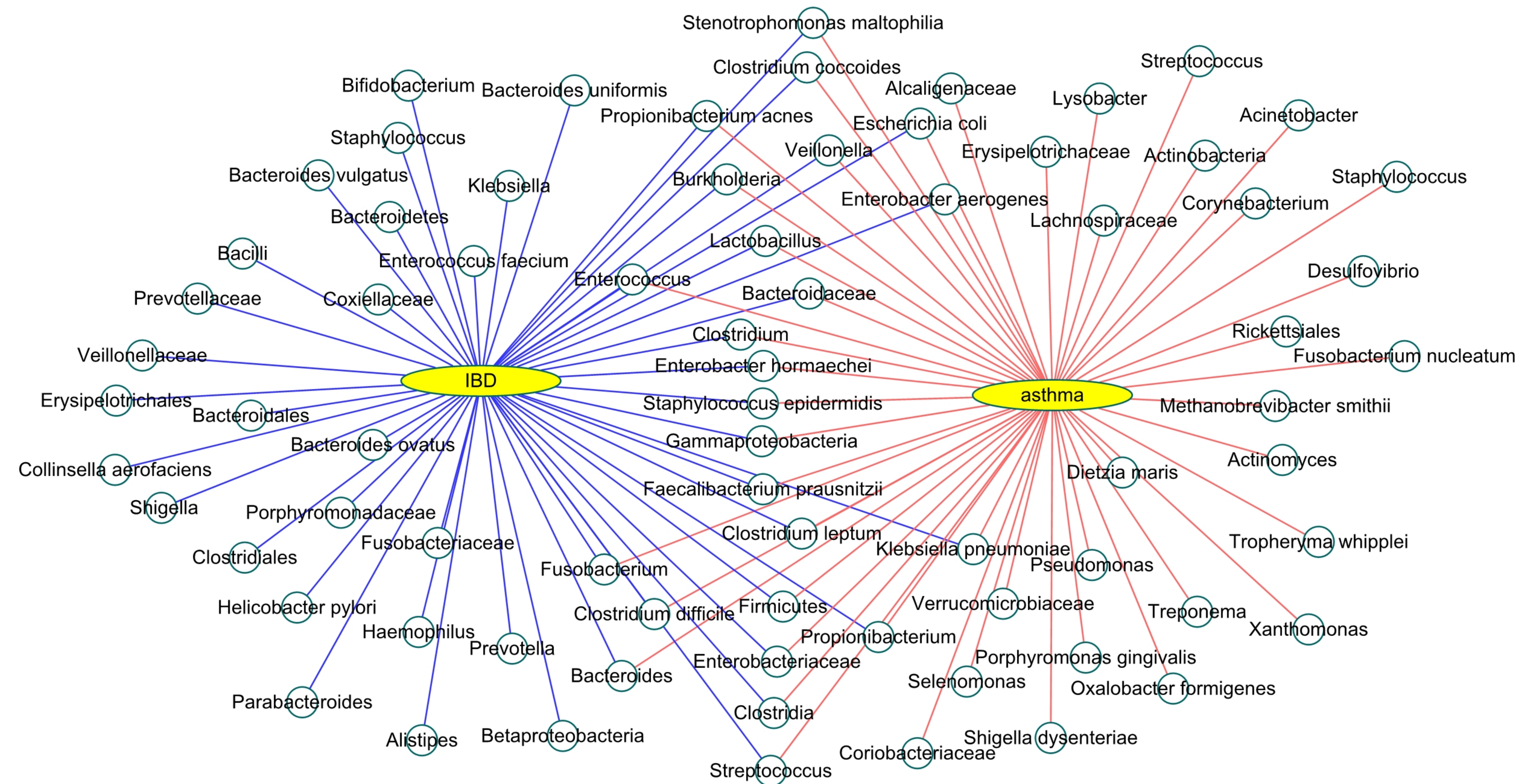

Supplement: Supplementary file 2 — Additional file 2 Figure S1. Network of the top-50 predicted associations for iBD and asthma obtained by our method. ellipses with Orange and circles represent diseases and microbes, respectively. the blue lines and red lines denote the associations of predicted microbes with iBD and asthma, respectively. [file 12859_2019_3066_MOESM2_ESM.pdf]
