# Supplementary material for: WMGHMDA: a novel weighted meta-graph-based model for predicting human microbe-disease association on heterogeneous information network
Source: BMC Bioinformatics. 2019 Nov 1;20:541. doi: 10.1186/s12859-019-3066-0 (PMC6824056; doi:10.1186/s12859-019-3066-0)
Supplement: Supplementary file 3 — Additional file 3 Figure S2. A simple example of how microbe functional similarity is calculated. [file 12859_2019_3066_MOESM3_ESM.pdf]

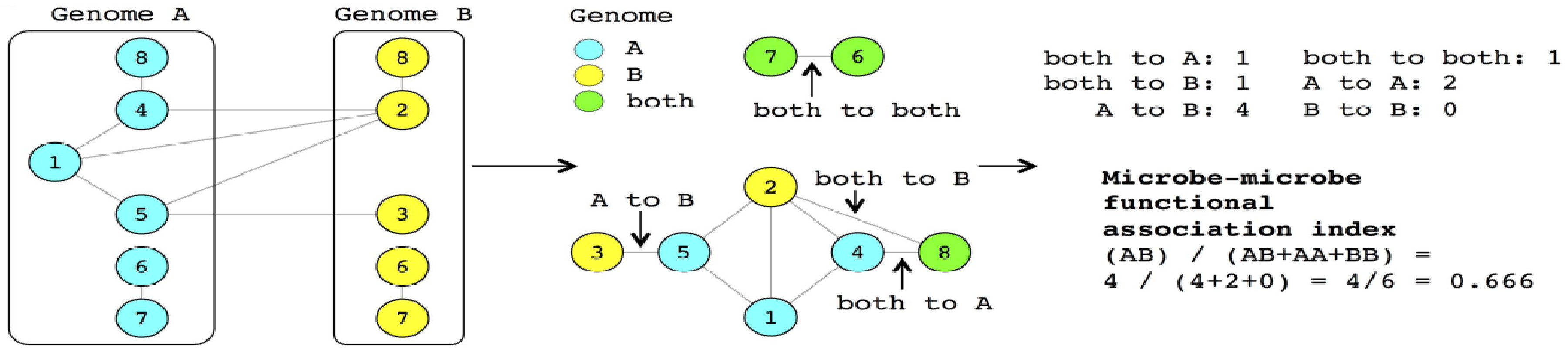

Fig. S2 A simple example of how microbe functional similarity is calculated. Genomes of two species (A and B) encode genes from 6 and 5 gene families respectively, three gene families are encoded exclusively in genome A (1, 4 and 5), two exclusively in genome B (2 and 3), and three in both genomes (6, 7 and 8). These three categories label the nodes of the protein functional association network. Edges connecting gene families are classified in 6 classes as shown on the figure. Edges connecting gene family encoded in only genome A to gene family encoded in only genome B would have to cross organismal boundary in order to exist within the network of two-species (A and B) community.
